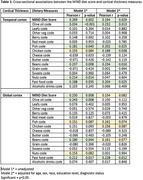# Association Between MIND Diet Score and Cortical Thickness in an Aging Population

**DOI:** 10.1002/alz.091779

**Published:** 2025-01-09

**Authors:** Desarae A. Dempsey, Liana G. Apostolova, Jared R. Brosch, David G. Clark, Martin R. Farlow, Sunu Mathew, Frederick Unverzagt, Sophia Wang, Sujuan Gao, Daniel Clark, Andrew J. Saykin, Shannon L. Risacher

**Affiliations:** ^1^ Stark Neurosciences Research Institute, Indiana University School of Medicine, Indianapolis, IN USA; ^2^ Indiana Alzheimer’s Disease Research Center, Indianapolis, IN USA; ^3^ Indiana University School of Medicine, Indianapolis, IN USA; ^4^ Department of Radiology and Imaging Sciences, Indiana University School of Medicine, Indianapolis, IN USA; ^5^ Department of Medical and Molecular Genetics, Indiana University School of Medicine, Indianapolis, IN USA; ^6^ Indiana University Network Science Institute, Bloomington, IN USA; ^7^ Department of Neurology, Indiana University School of Medicine, Indianapolis, IN USA; ^8^ Indiana University, Indianapolis, IN USA; ^9^ Regenstrief Institute, Inc, Indianapolis, IN USA; ^10^ Indiana University Center for Aging Research, Indianapolis, IN USA; ^11^ Indiana Alzheimer’s Disease Research Center, Indiana University School of Medicine, Indianapolis, IN USA; ^12^ Center for Computational Biology and Bioinformatics, Indiana University School of Medicine, Indianapolis, IN USA; ^13^ Center for Neuroimaging, Department of Radiology and Imaging Sciences, Indiana University School of Medicine, Indianapolis, IN USA

## Abstract

**Background:**

The Mediterranean diet has been associated with decreased brain atrophy (Staubo et al. 2016,*Alz&Dem*), but the MIND (Mediterranean‐Dietary Approaches to Stop Hypertension (DASH) Intervention for Neurodegenerative Delay) diet, designed for dementia prevention (Morris et al. 2015, *Alz&Dem*), remains underexplored for its impact on brain atrophy. We investigated the MIND diet’s association with cortical thickness (CT) in the Indiana Alzheimer’s Disease Research Center (IADRC) sample.

**Methods:**

134 participants (49 CN, 45 SCD, 30 MCI, 10 AD/other) completed a self‐report MIND diet questionnaire at the IADRC, which was coded into high, medium, or low intake groups for each food (5 ‘unhealthy’ food groups were reverse scored) and completed an MRI scan on a 3T scanner. The cortical surface was parcellated using FreeSurfer v6. We selected two regions of interest (ROIs) reflecting AD‐associated neurodegeneration: temporal and global CT. We examined the association of MIND diet scores (0‐15) and food groups with CT using regression models adjusted for age, sex, race, education, and diagnosis.

**Results:**

Higher MIND diet scores were associated with greater mean temporal CT (r = 0.269, p = 0.002) and greater mean global CT (r = 0.230, p = 0.008). In multivariable‐adjusted models, the association persisted for temporal but not global CT. Among the 15 food components, greater olive oil (r = 0.034, p<0.001), fish (r = 0.181, p = 0.040), beans (r = 0.237, p = 0.008), and nuts (r = 0.214, p = 0.014), and reduced fast food intake (r = 0.188, p = 0.035) were significantly associated with temporal CT. These associations, except for nuts, remained significant in multivariable‐adjusted models, with an additional relationship found for chicken (r = 0.189, p = 0.038). Among the 15 food components, greater olive oil (r = 0.243, p = 0.008), and beans (0.180, p = 0.044), and reduced fast food (r = 0.212, p = 0.017) were significantly associated with global CT. Only reduced fast food retained significance in the multivariable‐adjusted models.

**Conclusions:**

Greater adherence to the MIND diet was associated with greater CT in both global and temporal regions. Specific components, including increased olive oil, beans, nuts, fish, and reduced fast food, showed significant associations with CT, suggesting elements within the diet driving this association. These findings highlight the potential neuroprotective effects of the MIND diet, emphasizing the importance of dietary patterns in preserving brain health during aging.